# (2,2′-Bipyridine-κ^2^ *N*,*N*′)bis­(4-hydroxy­benzoato-κ^2^ *O*,*O*′)lead(II) monohydrate

**DOI:** 10.1107/S1600536810002941

**Published:** 2010-01-30

**Authors:** Jun Dai, Juan Yang

**Affiliations:** a Institute of Safety Science and Engineering, Henan Polytechnic University, Jiaozuo 454003, People’s Republic of China; b Department of Physical Chemistry, Henan Polytechnic University, Jiaozuo 454003, People’s Republic of China

## Abstract

The reaction of lead acetate, 4-hydroxy­benzoic acid and 2,2′-bipyridine in aqueous solution gave the title complex, [Pb(C_7_H_5_O_3_)_2_(C_10_H_8_N_2_)]·H_2_O. The asymmetric unit contains one Pb^II^ atom, two 4-hydroxy­benzoate ligands, one 2,2′-bipyridine ligand and one uncoordinated water mol­ecule. The Pb^II^ atom is hexa­coordinated in a distorted tetra­gonal-bipyramidal geometry and is chelated by four carboxyl­ate O atoms and two N atoms. O—H⋯O hydrogen-bonding inter­actions, involving the uncoordinated water, the carboxyl­ate O atoms and hydr­oxy O atoms, produce a three-dimensional supra­molecular structure.

## Related literature

For general background to the potential applications of lead compounds, see: Fan & Zhu (2006 ▶); Hamilton *et al.* (2004 ▶); Shi *et al.* (2007 ▶). For the use of aromatic carboxyl­ate ligands in the preparation of metal-organic complexes, see: Wang *et al.* (2006 ▶); Masaoka *et al.* (2001 ▶). For related lead structures, see: Shi *et al.* (2007 ▶).
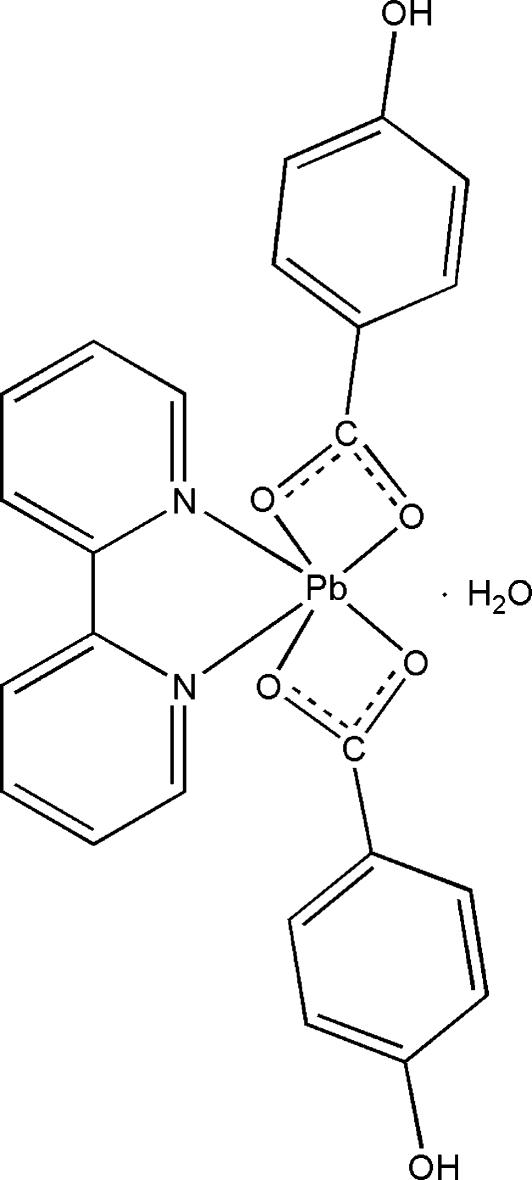


## Experimental

### 

#### Crystal data

[Pb(C_7_H_5_O_3_)_2_(C_10_H_8_N_2_)]·H_2_O
*M* *_r_* = 655.61
Monoclinic, 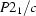

*a* = 10.9483 (4) Å
*b* = 17.5194 (6) Å
*c* = 12.0479 (4) Å
β = 100.334 (2)°
*V* = 2273.39 (14) Å^3^
*Z* = 4
Mo *K*α radiation
μ = 7.47 mm^−1^
*T* = 296 K
0.35 × 0.26 × 0.21 mm

#### Data collection

Bruker APEXII CCD area-detector diffractometer
Absorption correction: multi-scan (*SADABS*; Bruker, 2007 ▶) *T*_min_ = 0.180, *T*_max_ = 0.303
18618 measured reflections
4067 independent reflections
3579 reflections with *I* > σ(*I*)
*R*_int_ = 0.037

#### Refinement

*R*[*F* ^2^ > 2σ(*F* ^2^)] = 0.025
*wR*(*F* ^2^) = 0.072
*S* = 1.01
4067 reflections
315 parameters
3 restraints
H atoms treated by a mixture of independent and constrained refinement
Δρ_max_ = 1.05 e Å^−3^
Δρ_min_ = −0.61 e Å^−3^

### 

Data collection: *APEX2* (Bruker, 2007 ▶); cell refinement: *SAINT* (Bruker, 2007 ▶); data reduction: *SAINT*; program(s) used to solve structure: *SHELXS97* (Sheldrick, 2008 ▶); program(s) used to refine structure: *SHELXL97* (Sheldrick, 2008 ▶); molecular graphics: *SHELXTL* (Sheldrick, 2008 ▶); software used to prepare material for publication: *SHELXTL*.

## Supplementary Material

Crystal structure: contains datablocks global, I. DOI: 10.1107/S1600536810002941/pk2224sup1.cif

Structure factors: contains datablocks I. DOI: 10.1107/S1600536810002941/pk2224Isup2.hkl

Additional supplementary materials:  crystallographic information; 3D view; checkCIF report

## Figures and Tables

**Table 1:** Hydrogen-bond geometry (Å, °)

*D*—H⋯*A*	*D*—H	H⋯*A*	*D*⋯*A*	*D*—H⋯*A*
O6—H6*A*⋯O1^i^	0.82	1.90	2.671 (4)	157
O3—H3*A*⋯O1*W*	0.82	1.89	2.695 (5)	166
O1*W*—H1*A*⋯O5^ii^	0.90 (3)	2.04 (3)	2.849 (4)	148 (5)
O1*W*—H1*B*⋯O4^iii^	0.83 (3)	2.00 (3)	2.789 (4)	158 (5)

Symmetry codes: (i) 
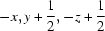
; (ii) 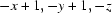; (iii) 
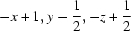
.

**Fig. 1.**
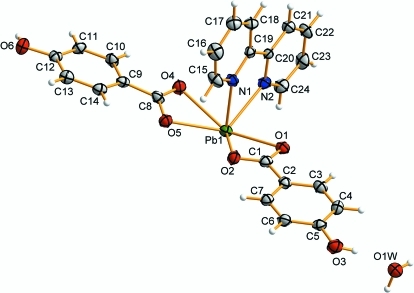
The coordination environment around PbII in the title complex with the atom-labeling scheme. Displacement ellipsoids for non-hydrogen atoms are drawn at the 30% probability level.

## supplementary crystallographic information

### Comment

Recently, lead compounds have been increasingly studied owing to their possible applications in different fields (Fan & Zhu 2006; Hamilton *et al.* 2004; Shi *et al.* 2007), such as ion-exchange, non-linear optics and catalysis. Environmental and biological concerns are due to the toxicity of lead and its diverse interactions with biological systems.  As an important family of multidentate O-donor ligands, aromatic carboxylate ligands have been extensively employed in the preparation of metal-organic complexes because of their potential properties and intriguing structural topologies (Wang *et al.* 2006; Masaoka *et al.* 2001). Herein, we report the structure of the title complex.

The asymmetric unit of the title complex, [Pb(C_24_H_18_O_6_N_2_)(H_2_O)], contains a Pb^II^ cation, two 4-hydroxybenzonic acid ligands, one 2,2'-bipyridine and one free water molecule, as illustrated in Fig. 1. The Pb^II^ atom is hexacoordinate and chelated by four carboxylate O atoms from two 4-hydroxybenzoic acid and two N atoms from one 2,2'-bipyridine. The Pb—O bond lengths are in the range of 2.477 (3) to 2.625 (3) Å. The Pb—N bond lengths are 2.463 (3) to 2.562 (3) Å. The Pb^II^ atom has a distorted tetragonal bipyramidal geometry. The free water molecule, carboxylate O atoms and hydroxy O atoms are involved in extensive O—H···O hydrogen-bonding interactions (Table 1).

### Experimental

A mixture of Pb(CH_3_COO)_2_ 3H_2_O (0.199 g, 0.52 mmol), 4-hydroxybenzoic acid (0.116 g, 0.84 mmol), 2,2'-bipyridine (0.033 g, 0.21 mmol) and distilled water (10 ml) was sealed in a 25 ml Teflon-lined stainless autoclave.  The mixture was heated at 403 K for 5 days to give colorless crystals suitable for X-ray diffraction analysis.

### Refinement

All H atoms bonded to C atoms and the hydroxy H atoms were placed in calculated positions (C—H = 0.93 Å, O—H = 0.82 Å) and were included in the refinement in the riding model approximation, with *U*_iso_(H) = 1.2Ueq(C) or *U*_iso_(H) = 1.5Ueq(O). The positions of the water H atoms were found in a difference Fourier map and refined with distance restraints O—H = 0.83 Å, *U*_iso_(H) = 1.5Ueq(O).

### Crystal data

**Table:**

[Pb(C_7_H_5_O_3_)_2_(C_10_H_8_N_2_)]·H_2_O	*F*(000) = 1264
*M**_r_* = 655.61	*D*_x_ = 1.915 Mg m^−^^3^
Monoclinic, *P*2_1_/*c*	Mo *K*α radiation, λ = 0.71073 Å
Hall symbol: -P 2ybc	Cell parameters from 9368 reflections
*a* = 10.9483 (4) Å	θ = 2.2–26.9°
*b* = 17.5194 (6) Å	µ = 7.47 mm^−^^1^
*c* = 12.0479 (4) Å	*T* = 296 K
β = 100.334 (2)°	Block, colorless
*V* = 2273.39 (14) Å^3^	0.35 × 0.26 × 0.21 mm
*Z* = 4	

### Data collection

**Table:**

Bruker APEXII CCD area-detector diffractometer	4067 independent reflections
Radiation source: fine-focus sealed tube	3579 reflections with *I* > σ(*I*)
graphite	*R*_int_ = 0.037
φ and ω scans	θ_max_ = 25.1°, θ_min_ = 2.2°
Absorption correction: multi-scan (*SADABS*; Bruker, 2007)	*h* = −13→13
*T*_min_ = 0.180, *T*_max_ = 0.303	*k* = −19→20
18618 measured reflections	*l* = −14→14

### Refinement

**Table:**

Refinement on *F*^2^	Primary atom site location: structure-invariant direct methods
Least-squares matrix: full	Secondary atom site location: difference Fourier map
*R*[*F*^2^ > 2σ(*F*^2^)] = 0.025	Hydrogen site location: inferred from neighbouring sites
*wR*(*F*^2^) = 0.072	H atoms treated by a mixture of independent and constrained refinement
*S* = 1.00	*w* = 1/[σ^2^(*F*_o_^2^) + (0.0468*P*)^2^ + 0.280*P*] where *P* = (*F*_o_^2^ + 2*F*_c_^2^)/3
4067 reflections	(Δ/σ)_max_ = 0.001
315 parameters	Δρ_max_ = 1.05 e Å^−^^3^
3 restraints	Δρ_min_ = −0.61 e Å^−^^3^

### Fractional atomic coordinates and isotropic or equivalent isotropic displacement parameters (Å^2^)

**Table:**

	*x*	*y*	*z*	*U*_iso_*/*U*_eq_	
Pb1	0.261796 (15)	0.580074 (8)	0.261115 (12)	0.03852 (9)	
O5	0.0806 (3)	0.64137 (15)	0.1348 (2)	0.0475 (7)	
C7	0.4017 (5)	0.4162 (2)	−0.0512 (4)	0.0420 (11)	
H7	0.3423	0.4505	−0.0863	0.050*	
C20	0.1550 (3)	0.4657 (2)	0.4545 (3)	0.0320 (8)	
O2	0.2797 (3)	0.50785 (17)	0.0880 (2)	0.0483 (7)	
N2	0.2459 (3)	0.51668 (19)	0.4497 (3)	0.0404 (8)	
O1	0.3906 (3)	0.45881 (15)	0.2420 (2)	0.0432 (7)	
C19	0.0708 (4)	0.4488 (2)	0.3465 (3)	0.0315 (8)	
C9	−0.0879 (4)	0.7114 (2)	0.1842 (3)	0.0356 (9)	
C2	0.4263 (4)	0.4108 (2)	0.0654 (4)	0.0375 (9)	
N1	0.0957 (3)	0.48437 (18)	0.2536 (2)	0.0380 (8)	
O6	−0.3930 (3)	0.85265 (16)	0.0985 (2)	0.0482 (7)	
H6A	−0.4058	0.8767	0.1536	0.072*	
C21	0.1424 (5)	0.4314 (2)	0.5547 (4)	0.0408 (10)	
H21	0.0775	0.3976	0.5575	0.049*	
C1	0.3605 (4)	0.4621 (2)	0.1352 (3)	0.0409 (10)	
C13	−0.2517 (4)	0.7641 (2)	0.0452 (3)	0.0419 (10)	
H13	−0.2914	0.7679	−0.0296	0.050*	
C14	−0.1501 (4)	0.7182 (2)	0.0728 (3)	0.0393 (9)	
H14	−0.1217	0.6909	0.0163	0.047*	
C3	0.5150 (4)	0.3590 (2)	0.1156 (3)	0.0462 (10)	
H3	0.5318	0.3543	0.1937	0.055*	
C10	−0.1360 (4)	0.7505 (2)	0.2682 (3)	0.0420 (10)	
H10	−0.0986	0.7452	0.3435	0.050*	
C12	−0.2965 (4)	0.8055 (2)	0.1294 (3)	0.0376 (9)	
O4	0.0747 (3)	0.65607 (16)	0.3160 (2)	0.0503 (8)	
C18	−0.0272 (4)	0.3996 (2)	0.3390 (4)	0.0424 (10)	
H18	−0.0429	0.3752	0.4035	0.051*	
C5	0.5550 (4)	0.3211 (2)	−0.0643 (3)	0.0399 (9)	
C16	−0.0779 (5)	0.4215 (2)	0.1430 (4)	0.0555 (13)	
H16	−0.1275	0.4132	0.0729	0.067*	
O3	0.6169 (3)	0.2796 (2)	−0.1324 (2)	0.0587 (8)	
H3A	0.6778	0.2594	−0.0940	0.088*	
C17	−0.1021 (4)	0.3859 (2)	0.2378 (4)	0.0504 (11)	
H17	−0.1691	0.3527	0.2332	0.061*	
C4	0.5782 (4)	0.3146 (2)	0.0512 (3)	0.0461 (10)	
H4	0.6371	0.2798	0.0859	0.055*	
C8	0.0289 (4)	0.6673 (2)	0.2142 (3)	0.0389 (9)	
C15	0.0219 (5)	0.4700 (2)	0.1548 (3)	0.0521 (12)	
H15	0.0393	0.4941	0.0907	0.063*	
C6	0.4647 (4)	0.3710 (2)	−0.1153 (3)	0.0466 (10)	
H6	0.4462	0.3740	−0.1936	0.056*	
C11	−0.2373 (4)	0.7962 (3)	0.2403 (3)	0.0447 (11)	
H11	−0.2677	0.8219	0.2971	0.054*	
C24	0.3265 (4)	0.5317 (2)	0.5439 (3)	0.0517 (11)	
H24	0.3901	0.5662	0.5400	0.062*	
C22	0.2272 (5)	0.4478 (2)	0.6510 (3)	0.0499 (11)	
H22	0.2208	0.4242	0.7188	0.060*	
C23	0.3208 (4)	0.4987 (3)	0.6463 (3)	0.0545 (12)	
H23	0.3786	0.5107	0.7104	0.065*	
O1W	0.8383 (3)	0.23234 (19)	−0.0179 (3)	0.0588 (8)	
H1A	0.885 (5)	0.273 (2)	−0.030 (4)	0.088*	
H1B	0.867 (5)	0.222 (3)	0.049 (3)	0.088*	

### Atomic displacement parameters (Å^2^)

**Table:**

	*U*^11^	*U*^22^	*U*^33^	*U*^12^	*U*^13^	*U*^23^
Pb1	0.03782 (13)	0.03792 (13)	0.04067 (12)	−0.00251 (6)	0.00932 (8)	0.00471 (6)
O5	0.0479 (19)	0.0526 (17)	0.0440 (15)	0.0115 (14)	0.0139 (14)	0.0043 (13)
C7	0.034 (3)	0.048 (3)	0.044 (2)	−0.0059 (18)	0.005 (2)	0.0071 (17)
C20	0.025 (2)	0.036 (2)	0.0352 (19)	0.0043 (16)	0.0062 (17)	0.0005 (15)
O2	0.0394 (18)	0.0588 (18)	0.0450 (15)	0.0114 (15)	0.0032 (14)	0.0044 (13)
N2	0.035 (2)	0.0451 (19)	0.0393 (17)	−0.0048 (16)	0.0027 (16)	0.0004 (14)
O1	0.0461 (19)	0.0453 (17)	0.0382 (15)	−0.0017 (14)	0.0077 (13)	0.0033 (12)
C19	0.031 (2)	0.0262 (18)	0.038 (2)	0.0034 (16)	0.0068 (17)	−0.0005 (15)
C9	0.042 (2)	0.031 (2)	0.0369 (19)	−0.0055 (17)	0.0141 (19)	0.0000 (15)
C2	0.037 (3)	0.037 (2)	0.039 (2)	−0.0053 (17)	0.0064 (19)	0.0038 (15)
N1	0.039 (2)	0.0410 (19)	0.0329 (16)	−0.0028 (15)	0.0029 (15)	0.0047 (13)
O6	0.0435 (19)	0.0505 (17)	0.0480 (16)	0.0107 (14)	0.0009 (14)	−0.0065 (13)
C21	0.042 (3)	0.041 (2)	0.039 (2)	−0.0018 (19)	0.007 (2)	0.0018 (17)
C1	0.035 (3)	0.043 (2)	0.045 (2)	−0.008 (2)	0.008 (2)	0.0026 (18)
C13	0.045 (3)	0.044 (2)	0.037 (2)	−0.004 (2)	0.0056 (19)	−0.0038 (17)
C14	0.045 (3)	0.037 (2)	0.037 (2)	−0.0042 (19)	0.0129 (19)	−0.0047 (16)
C3	0.050 (3)	0.047 (2)	0.040 (2)	−0.002 (2)	0.006 (2)	0.0025 (18)
C10	0.053 (3)	0.045 (2)	0.0287 (18)	0.006 (2)	0.0087 (19)	0.0018 (16)
C12	0.037 (2)	0.033 (2)	0.043 (2)	−0.0050 (18)	0.0082 (19)	−0.0024 (16)
O4	0.059 (2)	0.0521 (17)	0.0410 (16)	0.0137 (15)	0.0124 (15)	0.0015 (13)
C18	0.039 (3)	0.040 (2)	0.047 (2)	−0.0064 (19)	0.006 (2)	−0.0003 (18)
C5	0.031 (2)	0.051 (2)	0.0372 (19)	−0.0068 (19)	0.0069 (18)	−0.0010 (17)
C16	0.060 (4)	0.056 (3)	0.044 (3)	−0.010 (2)	−0.008 (2)	−0.0073 (19)
O3	0.055 (2)	0.076 (2)	0.0473 (16)	0.0091 (17)	0.0158 (16)	0.0009 (16)
C17	0.046 (3)	0.047 (3)	0.056 (3)	−0.010 (2)	0.001 (2)	−0.004 (2)
C4	0.041 (3)	0.046 (2)	0.050 (2)	0.009 (2)	0.006 (2)	0.0039 (19)
C8	0.051 (3)	0.036 (2)	0.0320 (19)	−0.0002 (19)	0.013 (2)	0.0014 (15)
C15	0.065 (3)	0.054 (3)	0.034 (2)	−0.014 (2)	−0.001 (2)	0.0032 (18)
C6	0.042 (3)	0.059 (3)	0.039 (2)	−0.006 (2)	0.009 (2)	0.0073 (19)
C11	0.049 (3)	0.047 (3)	0.043 (2)	0.006 (2)	0.019 (2)	−0.0029 (17)
C24	0.041 (3)	0.060 (3)	0.051 (2)	−0.010 (2)	−0.003 (2)	0.000 (2)
C22	0.060 (3)	0.055 (3)	0.033 (2)	0.001 (2)	0.003 (2)	0.0090 (19)
C23	0.047 (3)	0.068 (3)	0.040 (2)	−0.002 (2)	−0.013 (2)	0.001 (2)
O1W	0.060 (2)	0.060 (2)	0.0592 (19)	0.0064 (17)	0.0169 (18)	0.0124 (16)

### Geometric parameters (Å, °)

**Table:**

Pb1—N1	2.463 (3)	C13—H13	0.9300
Pb1—O2	2.477 (3)	C14—H14	0.9300
Pb1—O5	2.514 (3)	C3—C4	1.370 (6)
Pb1—N2	2.563 (3)	C3—H3	0.9300
Pb1—O1	2.583 (3)	C10—C11	1.359 (6)
Pb1—O4	2.625 (3)	C10—H10	0.9300
O5—C8	1.279 (4)	C12—C11	1.386 (5)
C7—C6	1.374 (6)	O4—C8	1.254 (4)
C7—C2	1.385 (6)	C18—C17	1.363 (6)
C7—H7	0.9300	C18—H18	0.9300
C20—N2	1.346 (5)	C5—O3	1.364 (5)
C20—C21	1.378 (5)	C5—C4	1.374 (5)
C20—C19	1.484 (5)	C5—C6	1.379 (6)
O2—C1	1.252 (5)	C16—C17	1.368 (6)
N2—C24	1.332 (5)	C16—C15	1.371 (6)
O1—C1	1.271 (5)	C16—H16	0.9300
C19—N1	1.351 (5)	O3—H3A	0.8200
C19—C18	1.366 (6)	C17—H17	0.9300
C9—C14	1.397 (5)	C4—H4	0.9300
C9—C10	1.402 (5)	C15—H15	0.9300
C9—C8	1.482 (6)	C6—H6	0.9300
C2—C3	1.387 (6)	C11—H11	0.9300
C2—C1	1.501 (6)	C24—C23	1.374 (6)
N1—C15	1.337 (5)	C24—H24	0.9300
O6—C12	1.341 (5)	C22—C23	1.368 (6)
O6—H6A	0.8200	C22—H22	0.9300
C21—C22	1.379 (6)	C23—H23	0.9300
C21—H21	0.9300	O1W—H1A	0.90 (3)
C13—C14	1.364 (6)	O1W—H1B	0.83 (3)
C13—C12	1.405 (5)		
			
N1—Pb1—O2	77.93 (10)	C13—C14—H14	119.3
N1—Pb1—O5	76.79 (10)	C9—C14—H14	119.3
O2—Pb1—O5	83.20 (9)	C4—C3—C2	120.7 (4)
N1—Pb1—N2	64.57 (10)	C4—C3—H3	119.7
O2—Pb1—N2	123.60 (11)	C2—C3—H3	119.7
O5—Pb1—N2	123.38 (10)	C11—C10—C9	120.3 (4)
N1—Pb1—O1	81.27 (11)	C11—C10—H10	119.9
O2—Pb1—O1	51.53 (9)	C9—C10—H10	119.9
O5—Pb1—O1	132.96 (8)	O6—C12—C11	123.3 (3)
N2—Pb1—O1	81.23 (9)	O6—C12—C13	118.6 (3)
N1—Pb1—O4	75.54 (10)	C11—C12—C13	118.2 (4)
O2—Pb1—O4	130.87 (9)	C8—O4—Pb1	91.5 (2)
O5—Pb1—O4	50.89 (8)	C17—C18—C19	120.6 (4)
N2—Pb1—O4	79.42 (10)	C17—C18—H18	119.7
O1—Pb1—O4	154.53 (9)	C19—C18—H18	119.7
C8—O5—Pb1	96.1 (2)	O3—C5—C4	122.9 (4)
C6—C7—C2	120.3 (4)	O3—C5—C6	117.6 (3)
C6—C7—H7	119.9	C4—C5—C6	119.5 (4)
C2—C7—H7	119.9	C17—C16—C15	117.7 (4)
N2—C20—C21	121.2 (4)	C17—C16—H16	121.1
N2—C20—C19	116.3 (3)	C15—C16—H16	121.1
C21—C20—C19	122.5 (4)	C5—O3—H3A	109.5
C1—O2—Pb1	95.8 (2)	C18—C17—C16	119.4 (4)
C24—N2—C20	118.4 (3)	C18—C17—H17	120.3
C24—N2—Pb1	122.0 (3)	C16—C17—H17	120.3
C20—N2—Pb1	119.6 (2)	C3—C4—C5	120.3 (4)
C1—O1—Pb1	90.4 (2)	C3—C4—H4	119.8
N1—C19—C18	120.6 (4)	C5—C4—H4	119.8
N1—C19—C20	116.6 (3)	O4—C8—O5	121.5 (4)
C18—C19—C20	122.7 (3)	O4—C8—C9	119.8 (3)
C14—C9—C10	118.1 (4)	O5—C8—C9	118.7 (3)
C14—C9—C8	122.0 (3)	N1—C15—C16	123.6 (4)
C10—C9—C8	119.9 (3)	N1—C15—H15	118.2
C7—C2—C3	118.7 (4)	C16—C15—H15	118.2
C7—C2—C1	120.2 (4)	C7—C6—C5	120.5 (4)
C3—C2—C1	121.1 (4)	C7—C6—H6	119.8
C15—N1—C19	118.1 (3)	C5—C6—H6	119.8
C15—N1—Pb1	119.0 (3)	C10—C11—C12	121.8 (4)
C19—N1—Pb1	122.8 (2)	C10—C11—H11	119.1
C12—O6—H6A	109.5	C12—C11—H11	119.1
C20—C21—C22	119.2 (4)	N2—C24—C23	123.5 (4)
C20—C21—H21	120.4	N2—C24—H24	118.2
C22—C21—H21	120.4	C23—C24—H24	118.2
O2—C1—O1	121.6 (4)	C23—C22—C21	119.9 (4)
O2—C1—C2	119.9 (4)	C23—C22—H22	120.1
O1—C1—C2	118.5 (4)	C21—C22—H22	120.1
C14—C13—C12	120.2 (4)	C22—C23—C24	117.8 (4)
C14—C13—H13	119.9	C22—C23—H23	121.1
C12—C13—H13	119.9	C24—C23—H23	121.1
C13—C14—C9	121.3 (4)	H1A—O1W—H1B	102 (4)

### Hydrogen-bond geometry (Å, °)

**Table:**

*D*—H···*A*	*D*—H	H···*A*	*D*···*A*	*D*—H···*A*
O6—H6A···O1^i^	0.82	1.90	2.671 (4)	157
O3—H3A···O1W	0.82	1.89	2.695 (5)	166
O1W—H1A···O5^ii^	0.90 (3)	2.04 (3)	2.849 (4)	148 (5)
O1W—H1B···O4^iii^	0.83 (3)	2.00 (3)	2.789 (4)	158 (5)

Symmetry codes: (i) −*x*, *y*+1/2, −*z*+1/2; (ii) −*x*+1, −*y*+1, −*z*; (iii) −*x*+1, *y*−1/2, −*z*+1/2.
